# Lifespan Based Pharmacokinetic-Pharmacodynamic Model of Tumor Growth Inhibition by Anticancer Therapeutics

**DOI:** 10.1371/journal.pone.0109747

**Published:** 2014-10-21

**Authors:** Gary Mo, Frank Gibbons, Patricia Schroeder, Wojciech Krzyzanski

**Affiliations:** 1 Department of Pharmaceutical Sciences, University at Buffalo, Buffalo, New York, United States of America; 2 DMPK Modeling and Simulation, Oncology, iMED, AstraZeneca, Waltham, Massachusetts, United States of America; Wayne State University School of Medicine, United States of America

## Abstract

Accurate prediction of tumor growth is critical in modeling the effects of anti-tumor agents. Popular models of tumor growth inhibition (TGI) generally offer empirical description of tumor growth. We propose a lifespan-based tumor growth inhibition (LS TGI) model that describes tumor growth in a xenograft mouse model, on the basis of cellular lifespan *T*. At the end of the lifespan, cells divide, and to account for tumor burden on growth, we introduce a cell division efficiency function that is negatively affected by tumor size. The LS TGI model capability to describe dynamic growth characteristics is similar to many empirical TGI models. Our model describes anti-cancer drug effect as a dose-dependent shift of proliferating tumor cells into a non-proliferating population that die after an altered lifespan *T_A_*. Sensitivity analysis indicated that all model parameters are identifiable. The model was validated through case studies of xenograft mouse tumor growth. Data from paclitaxel mediated tumor inhibition was well described by the LS TGI model, and model parameters were estimated with high precision. A study involving a protein casein kinase 2 inhibitor, AZ968, contained tumor growth data that only exhibited linear growth kinetics. The LS TGI model accurately described the linear growth data and estimated the potency of AZ968 that was very similar to the estimate from an established TGI model. In the case study of AZD1208, a pan-Pim inhibitor, the doubling time was not estimable from the control data. By fixing the parameter to the reported *in vitro* value of the tumor cell doubling time, the model was still able to fit the data well and estimated the remaining parameters with high precision. We have developed a mechanistic model that describes tumor growth based on cell division and has the flexibility to describe tumor data with diverse growth kinetics.

## Introduction

The integration of pharmacokinetic and pharmacodynamic (PK/PD) modeling in drug development has greatly improved the efficacy and safety of anti-cancer treatments. PK/PD models have allowed for better dose selection and optimized clinical trial designs. Recent efforts have demonstrated the benefits of applying PK/PD modeling in early stages of drug development. Advancement in PK/PD modeling, specifically the progression of PK/PD modeling from empirical to more mechanistic approaches have greatly increased the predictive power of models [1].

Empirical models are attractive because of their simplicity and parsimony, and for early compound screening based on specific criteria, they are very practical. The major drawback of empirical models is their reliance on drug-specific, rather than system-specific, parameters. Translation to higher species is expected to be challenging without knowledge of the biological system, since one would not know which parameters would change in a novel species. At the opposite end of the spectrum are mechanistic models of tumor growth, which combine drug-specific parameters with system-specific parameters for numerous molecular species. Although mechanistic models offer superior prediction accuracy, they often require rich datasets on numerous biomarkers in order to identify the parameters.

All tumor-growth inhibition (TGI) models have to be able to describe two hallmark characteristics of tumor growth and growth inhibition. Because tumor growth is a dynamic process, which can have profound effects on treatment success, accurate quantitative description of tumor growth is critical [2]. The unrestricted growth of solid tumors have been described using various mathematical functions (see [3], [4] for review). The onset of chemotherapeutic effect, namely inhibition of growth or reduction of tumor size, is often delayed [5]–[7]. This disconnect between the drug pharmacokinetics and the efficacy time course has been addressed in several TGI models [8]–[11]. Many mechanistic TGI models based on the integration of biological systems, both in tumor growth and anti-cancer treatment, have been developed [12]–[14]. These complex models offer great predictive potential, but all require additional biomarkers beside just tumor growth data.

The model developed by Simeoni and colleagues is described as a semi-mechanistic approach for modeling TGI [11]. The model is widely used in preclinical development due to its simplicity and features relevant to the biology of anti-cancer treatment. The model is able to capture both the complex growth kinetics and the temporal delay in tumor growth inhibition. However, the model still relies on empirical description of tumor growth, and offers no relevance to biological mechanisms that mediate growth of tumor cells.

The aim of this study is to develop a mechanistic TGI model that describes tumor growth through the process of cell division using the lifespan approach. Lifespan models are ideal for describing systems that have a finite duration, such as population of cells [15]. Drug-induced perturbation of biological systems is also well described using lifespan models [16], [17]. In our lifespan TGI model, the use of delay differential equations, which are developed to characterize delays in dynamic systems, offer a natural way of describing the delay in anti-cancer effect of drug treatment as have been previously reported [9]. Furthermore, using the lifespan approach to account for cellular division allows us to describe cell-cycle specific chemotherapeutics in a novel way.

In the following sections, we first review data collection, both *in vivo* and from literature. We introduce the lifespan concept, along with a cell-division efficiency parameter *p*, which will be central to our analysis. We describe a model of unperturbed tumor growth that is dependent on tumor size. We then extend this to perturbed tumor growth, with different drug mechanism of action, specifically non-cycle-specific drug effect model, and a cycle-specific model. After conducting sensitivity analysis on the models, we then apply them to three case studies of tumor growth inhibition by paclitaxel, AZ968 and AZD1208. We conclude the paper with a discussion of the results. We establish that this lifespan-based model has the versatility to describe TGI for anticancer drugs having various modes of action, and the robustness to estimate the required parameters from experimental data typical of a preclinical development program.

## Materials and Methods

### Literature Data Acquisition

Data used in Case Study 1 was obtained from mouse xenograft study reported by Simeoni and colleague [11]. To briefly summarize the study, mice bearing tumors derived from the HCT116 human colon carcinoma cell line were given intravenous (i.v.) injections of paclitaxel at 30 mg/kg every 4 days starting from day 8 after tumor inoculation. Data from the study was digitized using Graph Digitizer 2.0 (Nick's Production). Pharmacokinetics of paclitaxel was described using a two-compartment model outlined in the original report [11] and model parameters were fixed to *V* = 0.81 L/kg, *k_el_* = 0.868/h, *k_12_* = 0.006/h and *k_21_* = 0.0838/h.

### Animal Data

For Case Study 2 and 3, raw data from previous studies [18], [19] were obtained from the investigators. In brief, female NCr and CD1 mice (5–6 week old) were treated with AZ968, an anti-cancer agent that was synthesized by AstraZeneca R&D (Waltham, MA). HCT116 tumor cells (6×10^6^) were implanted by s.c. injection into the left flank of NCr mice. This is a human colon carcinoma line, commonly used in xenograft studies [20], [21]. Animals with established tumor xenografts, determined by tumor size reaching ∼150–200 mm^3^, were randomized and treated once daily with either vehicle (0.5% HPMC) or AZ968 by intraperitoneal (i.p.) injection with 10 to 15 mice per treatment group. Tumor volume was measured with calipers and calculated as tumor volume  =  (length × width^2^) ×0.5. Female NCr and CD1 mice.

For pharmacodynamic studies, mice with tumors of 150–200 mm^3^ were treated with either vehicle or AZ968, with three mice per dose. AZ968 or vehicle was administered once per week for three weeks, and AZ968 were administered at 10, 20 or 30 mg/kg. Tumor volume was assessed three times a week for three weeks with measurements taken on the day of treatment, 48 h and 120 h after treatment. For AZ968 treatment groups, an additional measurement was taken on 216 h after the last dose administration.

For pharmacokinetic study of AZ968, CD1 mice were given single i.p. injection of AZ968 at 10, 20 or 30 mg/kg with 3 mice per dose group. Blood samples were collected via cardiac puncture. Total plasma concentrations of AZ968 were determined by LC/MS/MS method. Same procedure was used for the pharmacokinetic study of AZD1208, except AZD1208 was administered orally (p.o.) at 3, 10 or 30 mg/kg doses as outlined in the original study [18].

### Model Development

#### Unperturbed tumor growth model

**Figure 1:**
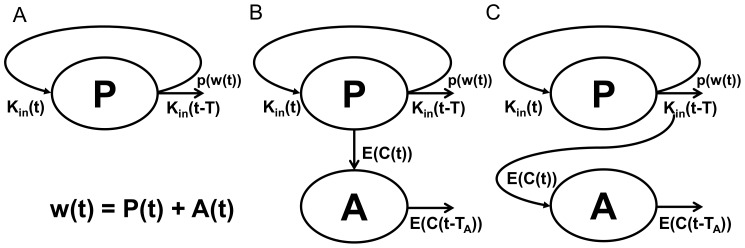
Model Schematics. A) Unperturbed tumor growth, B) Non-cycle-specific perturbed tumor growth, and C) Cycle-specific perturbed tumor growth. The meanings of the symbols and variables are explained in the model development section.



The schematic of the unperturbed tumor growth model is presented in Figure 1A. Lifespan models are similar to the widely used indirect response model [22] in that changes in the biological response of interest are governed by production and elimination [23]. In developing a lifespan formulation of tumor growth, we made the following assumptions: 1) Tumor size is controlled by two processes: production and elimination. 2) Each tumor cell has a lifespan *T*. When this time has elapsed, the cell divides in two, thus *T* is the tumor doubling time. 3) For simplicity, we assume all cells are at the same point of their lifespan. 4) Production of new tumor cells is exclusively due to division of existing cells. Under assumption (1), the net change in size is described by the following differential equation:
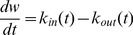
(eq. 1)where *k_in_(t)* and *k_out_(t)* are production and elimination rates, respectively. Under assumptions (2) and (3), the daughter cells produced from cell division become tumor cells and contribute to the growth of the tumor. Combined, these assumptions indicate that the lifespan of the tumor cells can be used to determine the elimination rate *k_out_(t)* from the production rate *k_in_(t)* using the following relationship [15]:

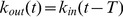
(eq. 2)


We assume that the production of new tumor cells is exclusively due to division of the cells. This allows us to propose the concept that changes in tumor growth rate with increasing tumor size is a result of changes in cell division efficiency, which results from a decrease in accessible nutrients as previously suggested [24]. In our model we incorporate division efficiency using the relationship:
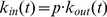
(eq. 3)


The dimensionless parameter *p* is the efficiency of cell division i.e., the number of cells that actually become new tumor cells. To ensure the tumor continues to grow *p* should not be less than 1, and under normal cytokinesis it cannot be greater than 2. Therefore, the division efficiency parameter is constrained by 1≤*p*≤2. This division efficiency parameter is central to our analysis and facilitates our theory that the tumor size impacts the efficiency of the division, and that tumor size limits growth by decreasing the efficiency of division of the tumor cells. Consequently, *p* must be a function that decreases with respect to tumor weight, *w*:
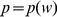
(eq. 4)


Finally, we assume that the model (eq. 1) applies for times *t*>0, where time *t* = 0 can be arbitrarily chosen to mark the beginning of the experiment, data collection, or treatment initiation. Since the full description of *k_out_(t)* requires knowledge of *k_in_(t)* for times *–T*<*t*≤0, we assume that the doubling time *T* is relatively small compared to the duration of the experiment design, and consequently the tumor production rate is relatively constant over the *–T*<*t*≤0 time interval:
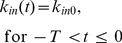
(eq. 5)


Based on the relationships outlined by eq. 2 and eq. 4, it is necessary to describe *k_in_(t)* using a recursive definition:
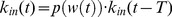
(eq. 6)


The derivation of the recursive form of *k_in_(t)* is discussed in Appendix S1, and *k_in_(t)* can now be presented as:
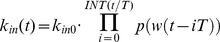
(eq. 7)where *INT(x)* denotes the biggest integer such that *INT(x)* ≤*x*. Please note that according to eq. 7, the number of delays is determined by the ratio *INT(t_last_/T)*, where *t_last_* is the last observation point or end of the time interval where the model applies. By combining the relationship outlined by eq. 7, eq. 2, and eq. 1 our model for unrestricted tumor growth can now be presented as:



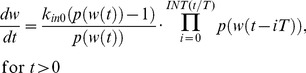
(eq. 8)To solve this equation uniquely, an initial condition is required, for which we choose: 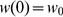
(eq. 9)where *w_0_* is the tumor size at time *t* = 0 i.e., the start of the experiment.

#### Constant efficiency p

Since this is a novel mathematical recasting of the problem, we examine some simplistic scenarios, to confirm that it behaves as one would expect. First let us take the case in which cell division efficiency is *not* affected by the tumor size. Under the assumption of constant division efficiency (p(w) ≡p_0_) eq. 8 simplifies to:
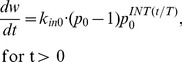
(eq. 10)which can be solved explicitly (see Appendix S2):



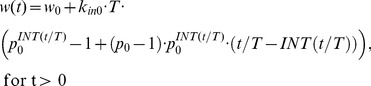
(eq. 11)Since 0≤*t/T - INT(t/T)* <1 one can obtain the lower and upper bounds for the solution that control the asymptotic behavior for large times:
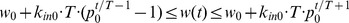
(eq. 12)


**Figure 2:**
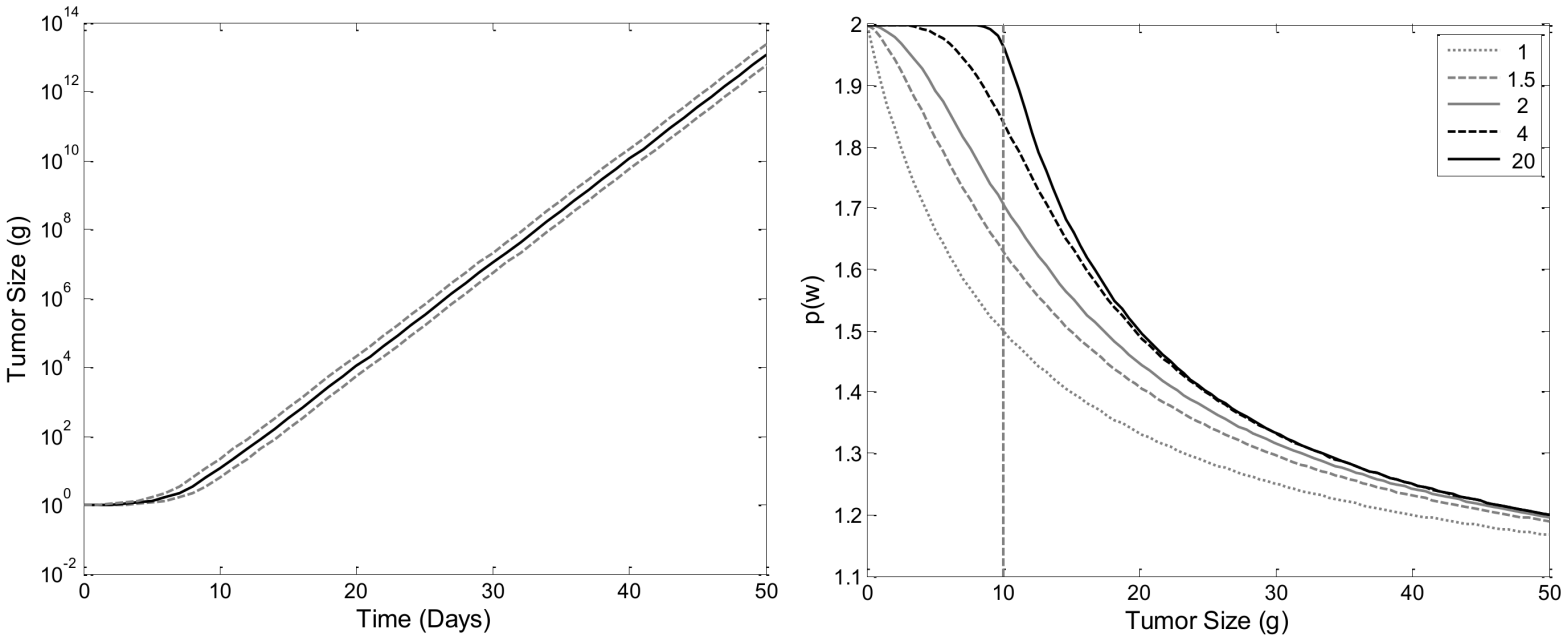
Simulated Effect of Division Efficiency on Tumor Growth. A) Semi-log plot of tumor growth with constant division efficiency of *p_0_* = 2. Upper and lower dashed lines indicate upper and lower bound described by eq. 12. B) Simulation of cell division efficiency function, *p(w)*, vs tumor weight, *w*, for different values of *ψ*. The threshold, *w_th_*, was set to 10 g, and initial efficiency, *p_0_* is set to 2.

It should be noted that because of the discontinuity of the function *INT(x)*, the exact asymptotic of *w(t)* for large *t* values might be difficult to obtain. However, for small lifespan *T* values the relationship (12) essentially says that *w(t)* grows exponentially with time as *p_0_^t/T^* which reinforces the interpretation of *T* as the time necessary to increase the tumor size by the factor *p_0_*. In the case of constant maximum division efficiency (*p_0_* = 2), then *T* becomes the exact doubling time of the tumor size. Figure 2A shows an example of the tumor growth curve described by eq. 11 with the upper and lower bounds (eq. 12).

#### Tumor size dependent efficiency

The data on growth of tumor xenograft in mouse models suggest an inverse relationship between the tumor size and the growth rate [25]. Numerous mathematical models have been developed to account for tumor size restriction of tumor growth rate using size-dependent inhibitory functions in model equations [11], [26], [27]. As outlined above, our approach is based on the assumption that the major impact of tumor size on its growth is on decreasing the efficiency of cell division. Therefore the function *p(w)* should decrease with increasing tumor weight, *w*. According to eq. 8, if for a specific tumor size *p(w_ss_)*  = 1, then tumor growth is stopped and *w_ss_* becomes the steady state solution. Alternatively, one can consider *p(w)* decreasing to 1 as *w* approaches infinity, then the steady sate is never reached which is a necessary condition of unlimited tumor growth. By design, most mouse xenograft experiments do not reach steady tumor volume, so we will focus on the later scenario. Another feature of xenograft tumor growth time course is a biphasic profile with an initial exponential growth followed by a slower linear phase [28]. The model by Simeoni and colleagues [11] addressed this phenomenon by introducing a threshold tumor size below which the tumor growth is exponential and above which it becomes linear. Similarly, we propose the following relationship between tumor size and efficiency of cell proliferation:
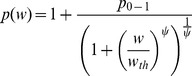
(eq. 13)where *p_0_* is the cell division efficiency for tumor sizes below the threshold *w_th_*. The power coefficient *ψ* serves as a continuous representation of a switch between exponential and non-exponential tumor growth phases. As *ψ*→∞, then:



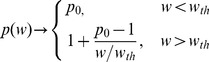
(eq. 14)Simulations of *p(w)* vs. *w* for a number of *ψ* values shown in Figure 2B demonstrate that for *ψ* = 20 the *p(w)* function exhibits a natural switch property (eq. 14).

**Figure 3:**
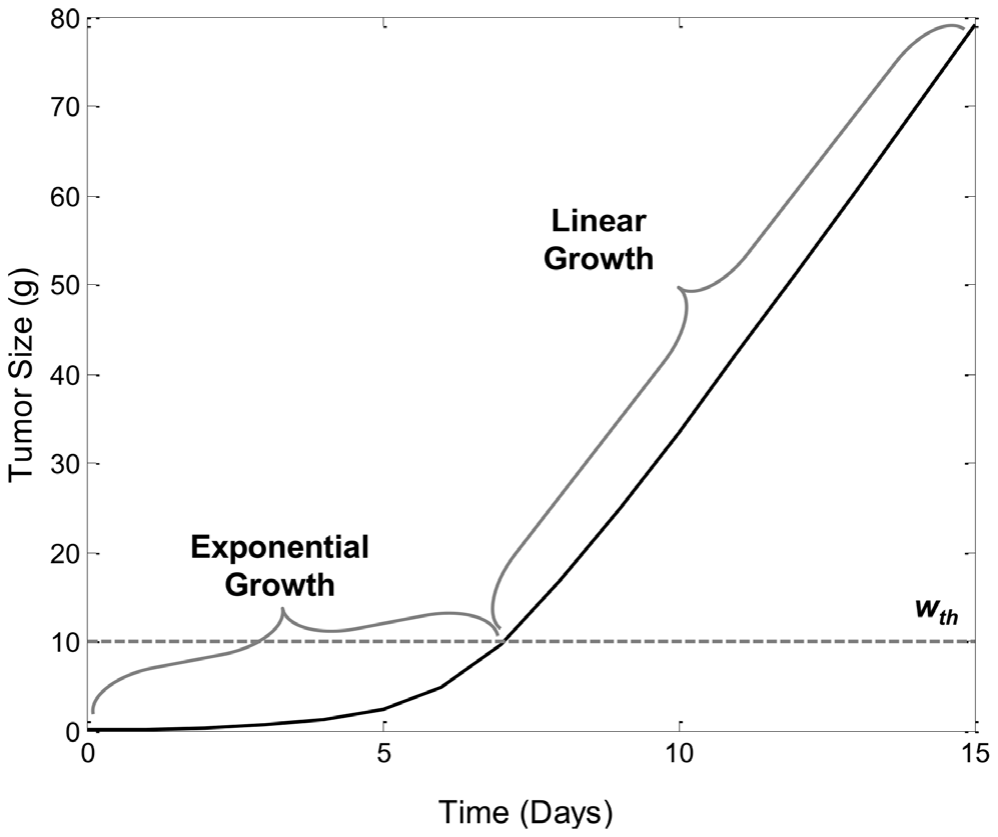
Simulation Tumor Growth Profile of TGI Lifespan Model. Plot of tumor weight vs. time. The threshold, *w_th_*, which is set to 10 g, is indicated by the dashed line. Doubling time of the tumor cells, *T*, was set to 1 day, *k_in0_* was set to 0.05 g/day and *p_0_* is set to 2.

In Appendix S3 we show that *w(t)* is an increasing function of time that approaches infinity as *t*→∞. Simulations of *w(t)* vs. *t* curves imply that for *w(t)*>*w_th_*, change in *w(t)* becomes linear (Figure 3). The calculation of the slope of this line is difficult. However, if we assume that *T* is small compared to other model time scales, then: 
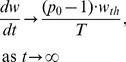
(eq. 15)


eq. 15 implies that the slope of the linear growth phase is proportional to *p_0_-1*, *w_th_*, and inversely proportional to *T*, but does not depend on *k_in0_* or *w_0_*.

If the tumor growth data exhibit only linear rate of tumor growth, we can assume that the measurements of tumor weight are taken after the tumor size has surpassed the threshold, such that the *w* observed are larger than the threshold value. Under such a scenario the *p(w)* function can be simplified to:
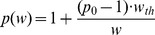
(eq. 16)which is derived from eq. 14. For the simplified form of the *p(w)* function (eq. 16), the parameters *p_0_* and *w_th_* are not individually identifiable, but the product *(p_0_ -1)·w_th_* can be consider as an identifiable model parameter, *p_wth_*, thus replacing the need to estimate *p_0_* and *w_th_*.

#### Perturbed tumor growth – non-cycle-specific drug effect model

The next phase of our model development is to introduce a model to account for anti-tumor drug induced tumor growth inhibition. We will first explore non-cycle-specific anti-cancer drugs that induce cell death (assumed to be apoptosis) in tumor during any stage of the tumor cell life cycle. The schematic representation of the non-cycle-specific drug effect model is presented in Figure 1B. Mathematical models of cytotoxic effect of anticancer agents relate drug plasma concentration *C(t)* to the rate of cell removal as second-order or saturable processes [10], [29], [30]. Because these anti-cancer drugs can affect the tumor cells at any time, the drug effects can be modeled simply as a dose-dependent removal of a portion of tumor cells from the replicating population. We assign such tumor cells to a non-proliferating population that will die of apoptosis. This requires the tumor to be separated into two populations of cells: a proliferating population *M(t)* and an apoptotic population *A(t)*, and sum of which makes up the tumor size:
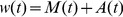
(eq. 17)


The process of removing the proliferating cells can be described as:
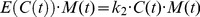
(eq. 18)which assumes a linear relationship between drug concentration, *C(t)*, and drug effect *E(C(t))*. The relationship is characterized by a second-order drug potency constant *k_2_*.

The killing of tumor cells affects proliferation in that only cells which survive the cytotoxic effects of the anti-cancer drug can divide at the end of their lifespan *T*. Consequently, the cell removal rate, *k_out_(t)* in our model is now presented as [16]:
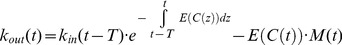
(eq. 19)where the integral multiplying *k_in_(t-T)* denotes the fraction of surviving cells. Given that only the surviving cells can divide with the efficiency *p(w(t))*, *k_in_(t)* is now presented as:



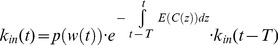
(eq. 20)Using the same recursive derivation presented in the Appendix S2, we can present *k_in_(t)* in the closed form:
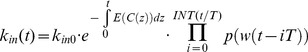
(eq. 21)


By combining eq. 19 and eq. 21 into eq. 1, the perturbed tumor growth by non-cycle-specific anti-cancer drugs can be expressed as:
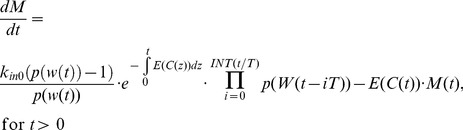
(eq. 22)


The initial condition for the proliferation population is:
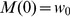
(eq. 23)


A hallmark feature of anti-cancer treatment is a significant time delay between plasma drug concentration and reduction of tumor size or inhibition of tumor growth [5]–[7], [9], [10], which have been addressed by various models using transit compartments [10], [11]. Similarly to the approach by Simeoni and colleagues [11], we assume that the cells affected by the drug are not killed instantaneously, but rather undergo programmed cell death (apoptosis) that takes a period of time *T_A_* to complete. If *A(t)* denotes the size of the apoptotic tumor cells due to a chemotherapeutic effect at time *t*, then according to the basic lifespan model [9], [15]:
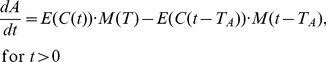
(eq. 24)


If no drugs were given prior to start of experiments then there is an absence of any non-proliferating tumor cells that are generated by anti-cancer drugs:
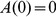
(eq. 25)


The perturbed tumor growth model described by eq. 22 and eq. 24 accounts for non-cycle-specific drug effects since the tumor cells are susceptible to drug-induced killing at any stage of their development at the rate specified by eq. 18. An example of the MATLAB implementation of the non-cycle-specific drug effect model is provided in Material S1–S3.

#### Perturbed tumor growth - cycle specific drug effect model

For this section we will address cycle-specific anti-cancer compounds that inhibit tumor growth by inducing apoptosis at a specific point of the tumor cell life cycle. The concepts for the cycle-specific drug effect model are generalized in the model schematic in Figure 1C. Since the turnover of tumor cells in our model is determined by their lifespan *T*, according to eq. 2, we will utilize another mechanism of drug action on tumor cells where the drug affects the lifespan distribution of the affected tumor cells [17]. 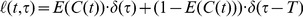
(eq. 26)


Here 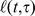 is the probability density function for the distribution of the cell lifespan *τ* at time *t*. The terms *δ(τ)* and *δ(τ-T)* are the point distributions (Dirac delta functions) centered at 0 and T, respectively, and the drug effect is described by the Emax model: 
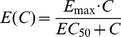
(eq. 27)with 0≤*E_max_*≤1 being the maximum effect, and *EC_50_* representing the drug concentration eliciting 50% of the maximum effect. According to eq. 26, the chemotherapeutic effect shifts cells of lifespan *T* to a subpopulation of cells with lifespan of 0, and the partition between these populations is determined by the Emax model. Since a cell of lifespan *T = *0 at a given time *t* must be immediately removed from the population, the drug effect results in instantaneous removal of portion of tumor cells from the population determined by the drug function *E(C(t))*. Based on concept [17], the cell elimination rate becomes:



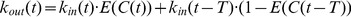
(eq. 28)The cells which are not affected by the chemotherapy become new tumor cells with efficiency *p(w(t))*, since the tumor weight now consists of both the proliferating cells *w(t)* and apoptotic cells *A(t)*. Thus, *k_in_(t)* must now be presented as:
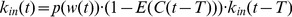
(eq. 29)


The relationship described in eq. 29 provides a recursive definition of *k_in_(t)* which leads to the following: 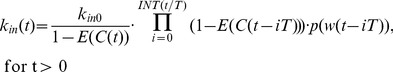
(eq. 30)


Consequently, as derived in Appendix S2, the perturbed tumor model becomes: 
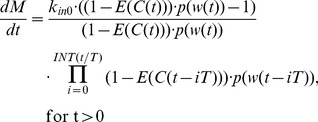
(eq. 31)with the initial condition described by eq. 9.

Similar to the non-cycle-specific drug effect model, the apoptotic cell population is described by the lifespan model [15]: 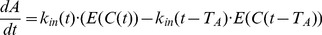
(eq. 32)with the initial condition given by eq. 25:

The model outlined by eq. 31 and eq. 32 describes drug action that is cycle-specific because the drug can only affect cells that have reached the end of their lifespan *T* when they divide, and tumor cells at any other stage of their development are not affected by the drug, which is the fundamental definition of cycle-specific anti-cancer drug effect [31]. An example of the MATLAB implementation of the cycle-specific drug effect model is provided in Material S4–S6.

### Data Analysis

All models in this report were implemented in MATLAB (R2012b, The MathWorks Inc.). Model parameter values were estimated using the function nlinfit, a nonlinear regression algorithm in MATLAB. The delay differential equations were solved using dde23 [32]. Unlike the other parameters, the lifespan TGI model is not a continuous function of *T* and *T_A_* due to the use of the integer function in eq. 8, which causes jumps in the model output over continuous value of *T*. Therefore, a grid search method was performed to estimate *T* and *T_A_*. Once the value of *T* and *T_A_* are determined, they were fixed and the remaining model parameters were estimated using nlinfit. Due to higher number of model parameters in perturbed lifespan TGI models, a grid search with all the parameters was not feasible in terms of run time. Instead, values of *T*, *p_0_*, *k_in0_* and *w_th_* were fixed to estimates obtained by fitting to the control group, *T* and *T_A_* were determined using grid search, and finally, remaining parameters were refitted with nlinfit, having fixed the values of *T* and *T_A_*.

For the comparison of the lifespan TGI model to the TGI model developed by Simeoni and colleagues [11], the tumor growth data from the AZ968 *in viv*o study outlined above were used. The data from that study only exhibit linear tumor growth, and are assumed to have surpassed the threshold size. Linear data can be accommodated in our lifespan TGI model using eq. 16 to describe division efficiency. For the reference TGI model, we have to make modification to the equation to only describe linear tumor growth data. The model equations are now presented as:
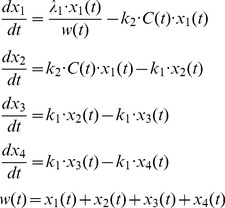
(eq. 33)where *λ_1_* is the linear growth rate, *k_2_* is the second order drug potency constant, *k_1_* is the transit rate constant, and *C(t)* is the plasma drug concentration.

## Results

### Model Exploration – Unperturbed Tumor Growth

The lifespan model of tumor growth inhibition outlined in the model development section accounts for tumor growth through the process of cellular division of tumor cells, and is capable of describing non-cycle-specific anti-cancer drug effects and cycle-specific drug effects. Exploration of the characteristics of our model will begin with the unperturbed tumor growth model. The incorporation of a tumor size-dependent cell division efficiency factor, *p(w(t))* as outlined by eq.13, allows the model to produce a bi-phasic growth kinetic with initial exponential growth rate and linear growth rate after a specific tumor size is reached (*w_th_*), as indicated by the model profile in Figure 3.

### Sensitivity Analysis – Unperturbed Tumor Growth

**Figure 4:**
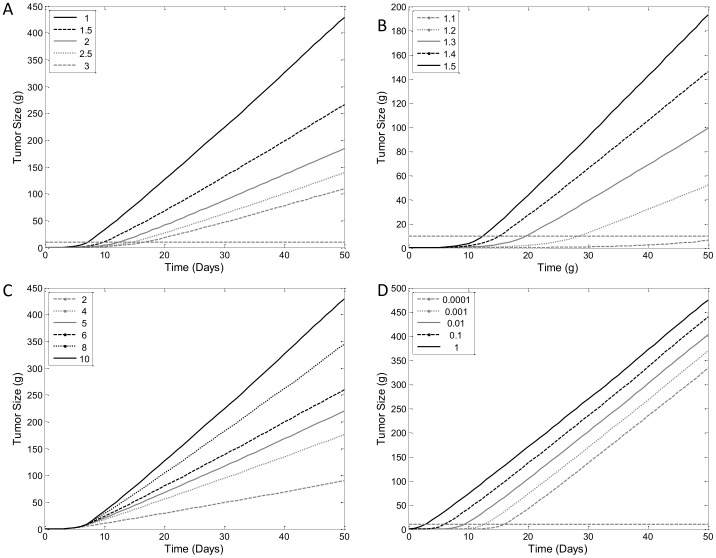
Sensitivity Analysis of Unperturbed Tumor Growth. Simulated model profiles of varying values of A) Tumor cell lifespan (*T*), B) Initial division efficiency (*p_0_*), C) Tumor size threshold (*w_th_*) for decrease in division efficiency, and D) Past tumor growth rate (*k_in0_*). Simulation were carried with parameters values of *T* = 1 day, *p_0_* = 2, *k_in0_* = 0.05 g/day and *w_th_* = 10 g, unless otherwise specified for each figure. Dashed lines in panels A–C indicate the *w_th_* value.

The model parameters that determine the aspects of the model profile, such as slope of linear growth phase, have been explored mathematically in the model development section. Here we will further examine the effects of the parameters. The sensitivity analysis demonstrates that each of the model parameters has different effects on model behavior (Figure 4). Increasing values of *T* resulted in slower growth kinetics. Both exponential growth rate and linear growth rate (slope of linear growth phase) are affected (Figure 4A). Decreasing values of *p_0_* had a similar effect as increasing value of *T*, except *p_0_* tends to affect exponential growth much more than the linear growth (Figure 4B). Increasing values of *w_th_* does not have any effect on exponential growth, but does dictate the end of the exponential growth phase and more importantly the slope of the linear growth phase (Figure 4C). The effect of *T*, *p_0_*_,_ and *w_th_* on linear growth as seen in the sensitivity analysis is in accordance to the relationship of these parameters in determining the slope of linear growth into later time points as outlined by eq.15. Unlike the other parameters, changes in *k_in0_* do not seem to affect the kinetics of tumor growth in either phases, but rather determine when measureable level of growth begins (Figure 4 D). The results of the sensitivity analysis indicate that parameters in the unperturbed models are all identifiable. Confirmation of identifiability will be presented in the case studies with TGI data.

### Sensitivity Analysis – Non-Cycle-Specific Drug Effect

**Figure 5:**
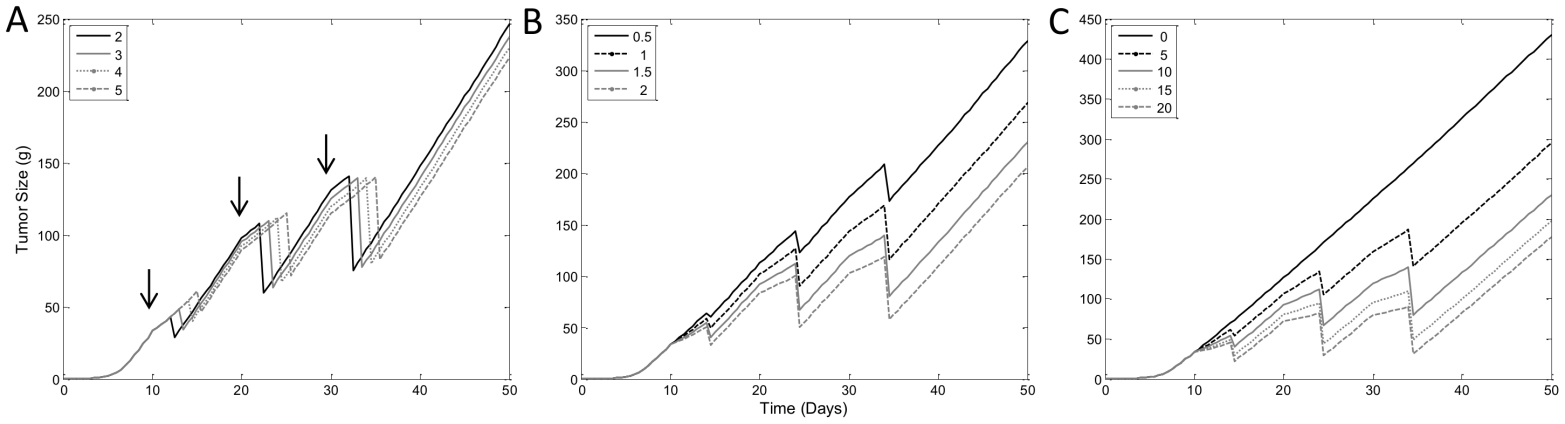
Sensitivity Analysis of Non-Cycle-Specific Drug Effect Model. Simulated model profiles with changes in A) Apoptosis duration, *T_A_*, and B) Linear drug potency constant, *k_2_*. C) Signature profile of cycle-specific drug mechanism model with dose escalation. Simulation were carried with parameters values of *T* = 1 day, *T_A_* = 4 days, *p_0_* = 2, *k_in0_* = 0.05, *w_th_* = 10 g, and *k_2_* = 1.5 mL/ng, unless otherwise indicated. Arrows indicate dose administration on days 10, 20 and 30.

Simulations for sensitivity analysis and model signature profiles were conducted with a two- compartment PK model with *k_el_* = 20 day^−1^, *k_12_* = 0.2 day^−1^, *k_21_* = 2 day^−1^ and *V* = 1 mL. For sensitivity analysis, a dose of 10 units was administered at 10, 20 and 30 days (we take dose to be dimensionless). Simulations of changes in parameters for the non-cycle-specific drug effect model were performed and the resulting sensitivity analysis is shown in Figure 5. Simulation shows that changes in the duration-of-apoptosis parameter, *T_A_*, have a direct effect on the length of delay of drug-induced tumor reduction after drug administration (Figure 5A). Changes in the linear drug potency constant, *k_2_*, affect the degree of TGI and tumor size reduction, if any (Figure 5B). Signature profile of the non-cycle-specific drug effect model with increasing doses of anti-cancer drug is shown in Figure 5C, and as expected is very similar to the simulation of changing values of *k_2_*.

#### Sensitivity analysis – cycle-specific drug effect

**Figure 6:**
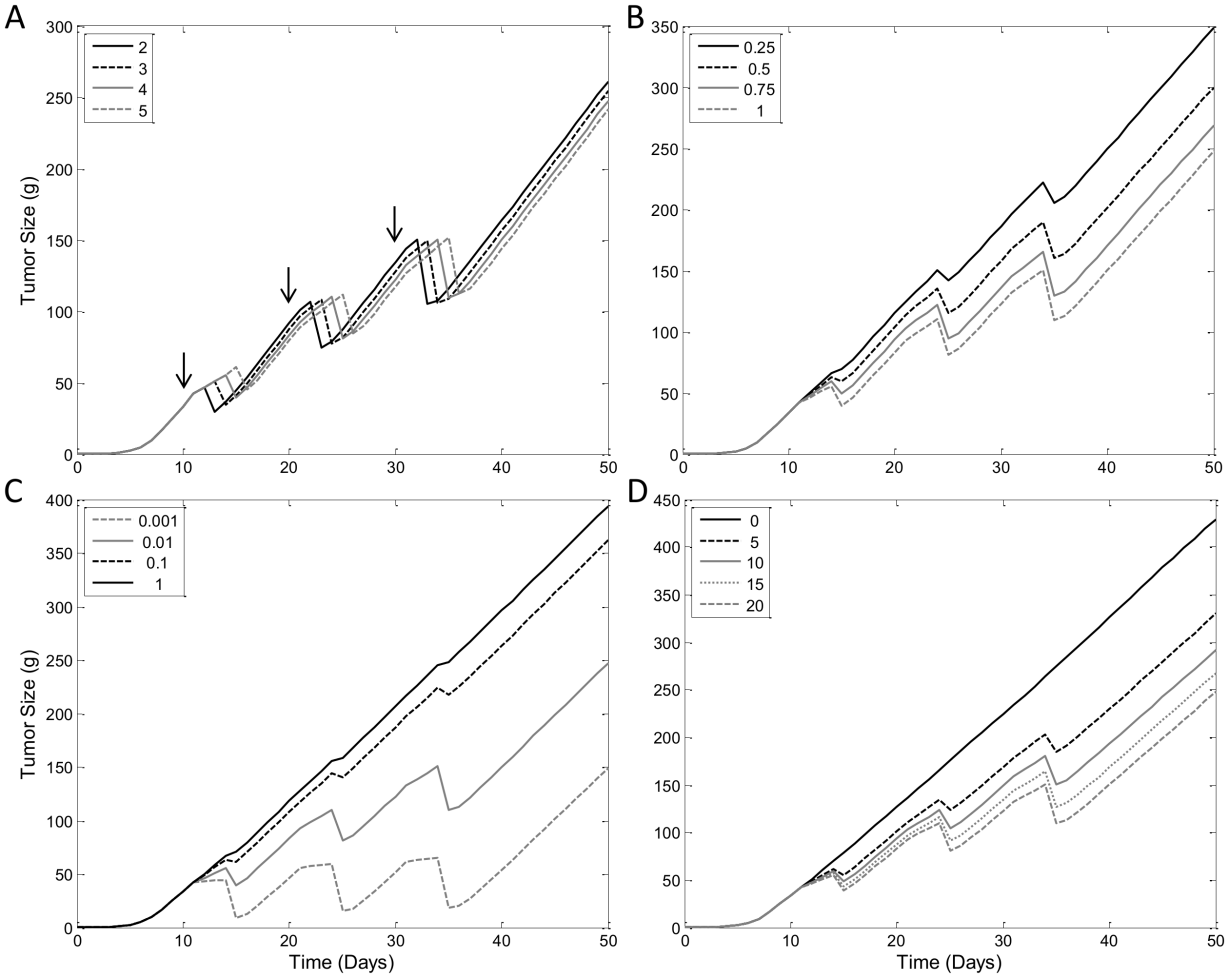
Sensitivity Analysis of Cycle-Specific Drug Effect Model. Simulated model profiles with changes in: A) Apoptosis duration, *T_A_*, B) Maximum drug efficacy, *E_max_*, and C) Drug potency, *EC_50_*. D) Signature profile of cycle-specific drug mechanism model with dose escalation. Simulation were carried with parameters values of *T* = 1 day, *T_A_* = 4 days, *p_0_* = 2, *k_in0_* = 0.05 g/day, *w_th_* = 10 g, *E_max_* = 1 and *EC_50_* = 0.01 concentration, unless otherwise indicated. Arrows indicate dose administration at days 10, 20 and 30.

Although both the cycle-specific and non-cell cycle-specific drug effect models use the same tumor growth component as the non-cycle-specific drug effect model, there are significant differences between them, as outlined in the Model Development section. Furthermore, unlike the non-cycle-specific model, which uses a linear drug effect function, the cycle-specific model incorporates the Emax model for drug effect and different parameters are required. Similar to sensitivity analysis of the non-cycle-specific model, changes in *T_A_* parameter exclusively affect the delay in anti-cancer drug effect (Figure 6A). The Emax drug effect requires both maximum efficacy, *E_max_*, and drug potency, *EC_50_*. Simulations show that changes in *E_max_* and *EC_50_* both affect the degree of anti-cancer drug induced tumor reduction (Figure 6B,C). Although similar, there are subtle differences in the effects of the two parameters that can be distinguished given the appropriate dose ranges in data. Notice the low *EC_50_* values used for simulation in Figure 6B (Figure legend), when compared to the peak concentration of the drug in the system which is 10 units/mL. This is due to the nature of the equation for the cycle-specific drug effect model which only allows the drug to act on tumors cell the moment they reach the doubling time, *T*. This small window means the drug can only affect a fraction of the cells. The model has to adjust the potency of the drug to account for efficacy. For this reason a much lower *EC_50_* value is required to account for the observed drug effect on tumor size. The effect of this feature is seen in the signature profile of the cycle-specific drug effect model shown in Figure 6D. Note the low degree of dose-separation for the effect of drug on tumor size. A low *EC_50_* value results in saturation of drug effect for all doses, prolonging the duration, but not the intensity, at higher doses. The effects of this model characteristic will be further evident in the following case study.

### Case Study 1 - Tumor Growth Inhibition by Paclitaxel

**Figure 7:**
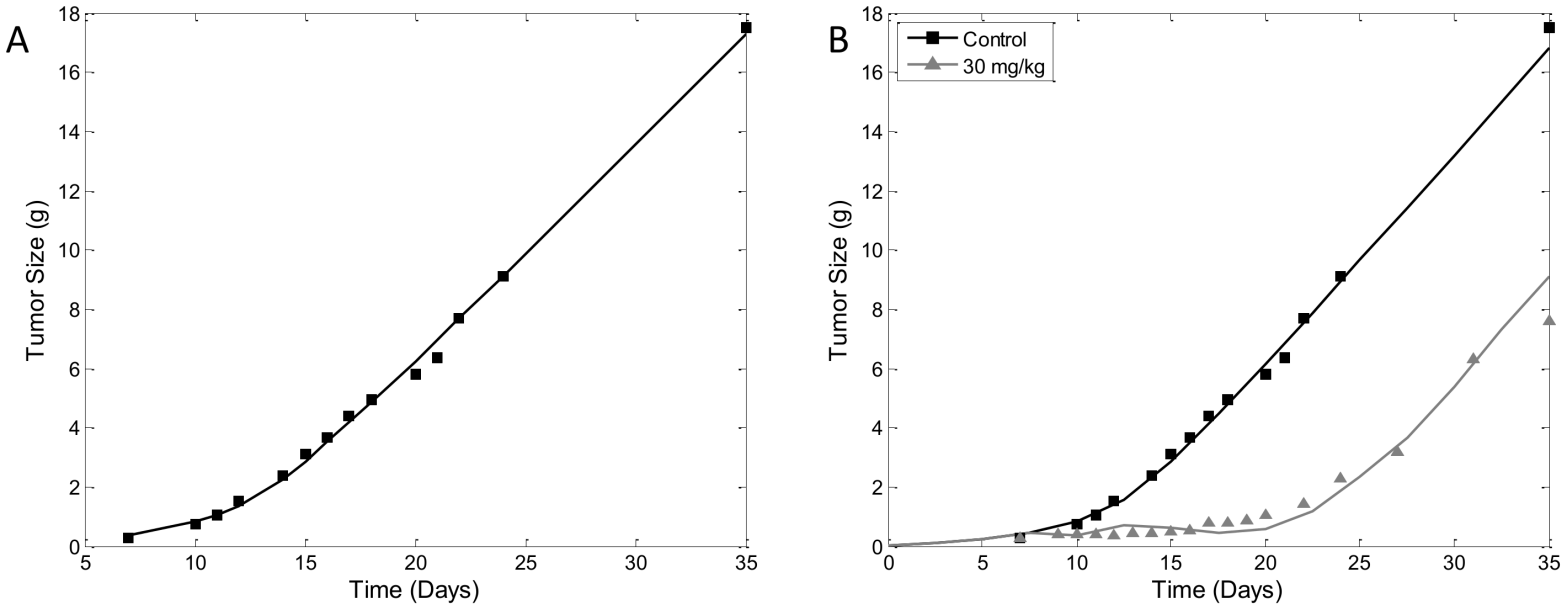
Modeling Tumor Growth Inhibition by Paclitaxel. A) Observed (black squares) and model fitted (black line) tumor weight during untreated tumor growth. Data was digitized from (Simeoni et al, 2004). Initial tumor volume was fixed at 0.033 g as estimated from original publication. B) Simultaneous fitting of unperturbed tumor growth data (black square) with model preduction (black line) and tumor growth inhibition data (grey triangle) and model prediction using the cycle-specific drug effect model (grey line) by 30 mg/kg of paclitaxel administered every 4 days for 3 rounds starting from day 8.

Data for this case study was obtained from literature [11]. In this study paclitaxel was administered i.v. at 30 mg/kg every 4 days starting from day 8 after tumor inoculation. Tumor growth data and model prediction by our lifespan TGI are shown in Figure 7. We began by fitting the unperturbed growth data. Prediction from our lifespan TGI model overlapped well with the experimental data (Figure 7A). Examination of the parameter estimates shows biologically relevant values (Table 1). The doubling time, *T*, was estimated to be 1.46 days, initial division efficiency, *p_0_*, estimated to be 1.44, past tumor production rate, *k_in0_*, was estimated to be 0.0386 g·day^−1^, and tumor threshold, *w_th_*, to be 2.55 g. The precision of the parameter estimates are high for both *p_0_* and *w_th_*, which had coefficient of variance (CV%) of 7.44% and 24.3%. The precision for the estimate of *k_in0_* was not as high as the other two, but was still within acceptable levels of precision (CV% of 77.8%).

**Table 1:** Parameter Estimates for Unperturbed Tumor Growth.

Parameter	Estimates	Units	CV%
*T*	1.46^a^	days	-
*p_0_*	1.44	-	7.44
*k_in0_*	3.86×10^−2^	g/day	77.8
*w_th_*	2.54	g	24.3

a Parameter was fixed.

For the second part of this case study we fitted both unperturbed and paclitaxel inhibited tumor growth data. Because paclitaxel is considered a cycle-specific anti-cancer drug, we fitted the data using the cycle-specific drug effect model. Figure 7B shows the experimental data and lifespan TGI model predicted values, which overlap well. Examination of the model parameter estimates show that values from the simultaneous fit were very similar to the estimates from the unperturbed data (Table 2). Estimate for *p_0_* was 1.44, *k_in0_* was 0.404 g day^−1^ and *w_th_* was 2.461 g. The estimate of *T* was kept constant from the result of the grid search from the unperturbed data fitting. Parameters unique to the cycle-specific drug effect model are *T_A_*, *EC_50_* and *E_max_*. *EC_50_* was estimated to be 83.28 ng/mL, *E_max_* was fixed to 1 due to lack of escalating doses, and *T_A_*, which also has discontinuous property similar to *T*, was estimated to be 0.536 days. The advantage of the simultaneous fitting is the availability of more data points. As expected the precision of the model estimates was increased compared to fitting only unperturbed data. CV% value of *p_0_*, *k_in0_*, *w_th_* and *EC_50_* were 0.0744%, 0.0908%, 2.225% and 0.043%, respectively. This case study demonstrates that our model is fully capable of fitting real experimental data from animal xenograft models. Interestingly, the estimated *EC_50_* value is very low compared to the peak plasma concentration of paclitaxel, which can reach 37040 ng/mL (data not shown), according to the PK model. Similar to the sensitivity analysis of the cycle-specific drug effect model, this observation is due to the limited time that a fraction of the tumor cells will be affected by the drug. Implication of this model characteristic will be discussed further in the following section. Regardless of the unique characteristic of the model, the high precision of the parameter estimates confirms the identifiability of the model parameters in the unperturbed model of tumor growth and the cycle-specific drug effect model. However, interpretation of the model parameters must be done with care, and adjustments of the model may be required, which will be discussed in the following section.

**Table 2:** Parameter Estimates for Simultaneous Fitting of Unperturbed and Paclitaxel Inhibited Tumor Growth with Cycle-Specific Drug Effect.

Parameter	Estimates	Units	CV%
*T*	1.46^a^	days	-
*T_A_*	0.536^a^	days	-
*p_0_*	1.44	-	2.18×10^−2^
*k_in0_*	4.04×10^−2^	g/day	0.149
*w_th_*	2.48	g	2.24
*E_max_*	1^a^	-	-
*EC_50_*	9.45	ng/mL	2.23

a Parameter was fixed.

### Model Comparison – Tumor Growth Inhibition by AZ968

**Figure 8:**
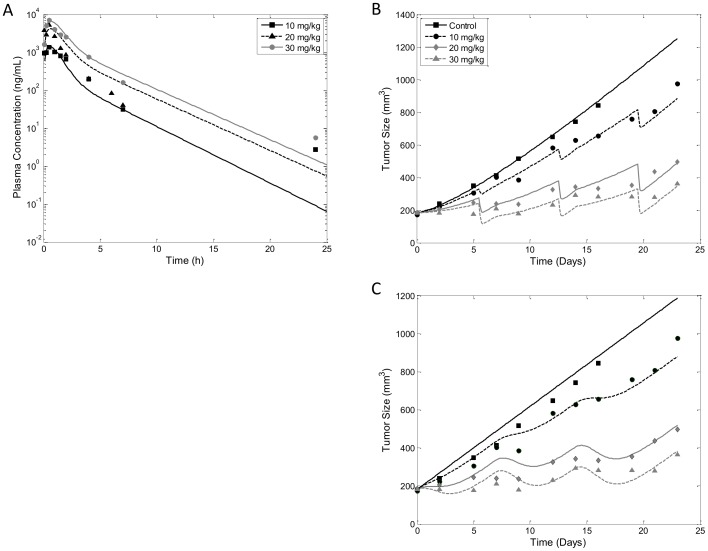
Model Comparison: Modeling Tumor Growth Inhibition by AZ968. A) Modeling of pharmacokinetic data after single i.p. dose of AZ968 at 10, 20 and 30 mg/kg. Data (symbols) was described with a 2 compartment model with dose-dependent elimination rate constant and central volume of distribution (model prediction in lines). B) Observed (symbols) and model predicted (lines) tumor volume using the lifespan model of tumor growth inhibition. Line style and color indicate unrestricted condition and oral treatment with AZ968 at 10, 20 and 30 mg/kg in mice xenograft. Symbols indicate control condition (black squares), 10 mg/kg AZ968 (black circles), 20 mg/kg (grey diamonds), and 30 mg/kg (grey triangles). Initial tumor volume was fixed at 180 mm^3^ as estimated from initial data points. C) Fitting of same AZ968 data using the Simeoni model.

Now that we have demonstrated the flexibility of our lifespan TGI model, and confirm identifiability of the model parameters, we will explore the capability of our model to describe mouse xenograft data compared to the TGI model presented by Simeoni and colleagues [11]. We first modeled the PK profile of AZ968, using a two-compartment model. Model fitting is shown in Figure 8A. Estimates of model parameter values are listed in Table 3. Due to observed non-linear PK, dose-specific values of *k_el_* and *V* were needed to fit PK data (Table 3). Unlike the paclitaxel datasets, data from this study show only linear growth kinetics. We will make the assumption that the tumor sizes at the first measurement have already surpassed the tumor threshold. The data will be modeled using the linear version of the lifespan TGI model (see eq. 16), and the linearized version of the Simeoni, et al., model of TGI (see eq. 33). AZ968 is a potent casein kinase 2 (CK2) inhibitor, which induces apoptosis of tumor cells. There is no known mechanism of AZ968 to suggest the compound is active only at specific cell cycles, therefore the non-cycle-specific drug effect model is used for this study. Model fitting of both the lifespan TGI model and the reference TGI model (eq. 33) are shown in Figure 8. Predictions from both models overlay well with the data points, and both models are able to capture the delayed onset of drug effect on tumor size. Parameter estimates for both model fittings are listed in Table 4. While the estimated value corresponding to ‘time till cell death’ (*T_A_* = 5.56 days for our model, and mean transit time (4/*k*_1_) = 2.68 days for Simeoni's) are not identical, they are comparable. The potency constant, *k_2_*, from the two models is estimated to be very similar: the lifespan TGI model gives *k_2_* = 0.0023 ng^−1^ mL·day^−1^ and the Simeoni model *k_2_* = 0.0020 ng^−1^·mL·day^−1^. Examination of the precision of the model estimates demonstrates that both models were able to estimate the parameters with high precision (see Table 4). This comparative study demonstrates that the lifespan TGI model is fully capable of describing real experimental data with precision comparable to that of one of the most commonly used models of TGI.

**Table 3:** Parameter Estimates for PK Fitting of AZ968.

Parameter	Estimates	Units	CV%
*k_a_*	46.8^a^	1/day	-
*k_el_* 10 mg/kg	33.9	1/day	1.40
*k_el_* 20 mg/kg	26.0	1/day	1.40
*k_el_* 30 mg/kg	24.7	1/day	1.40
*k_12_*	8.74	1/day	3.44
*k_21_*	11.2	1/day	0.801
*V* 10 mg/kg	2.70	L/kg	1.49
*V* 20 mg/kg	2.07	L/kg	1.49
*V* 30 mg/kg	1.97	L/kg	1.49

a Parameter was fixed.

**Table 4:** Parameter Estimate for Model Fitting of Tumor Growth Inhibition by AZ968.

Lifespan TGI Model				Simeoni TGI Model			
Parameter	Estimates	Units	CV%	Parameter	Estimates	Units	CV%
*T*	1.28^a^	days	-	*λ_1_*	43.8	1/day	6.98×10^−2^
*T_A_*	5.56^a^	days	-	*k_1_*	1.12	1/day	4.27×10^−2^
*P_wth_*	80.8	mm^3^	2.65	*k_2_*	2.0×10^−3^	mL/ng/day	0.101
*k_in0_*	49.9	mm^3^/day	4.46				
*k_2_*	2.30×10^−3^	mL/ng/day	3.69×10^−2^				

a Parameter was fixed.

### Case Study 2 - Tumor Growth Inhibition by AZD1208

**Figure 9:**
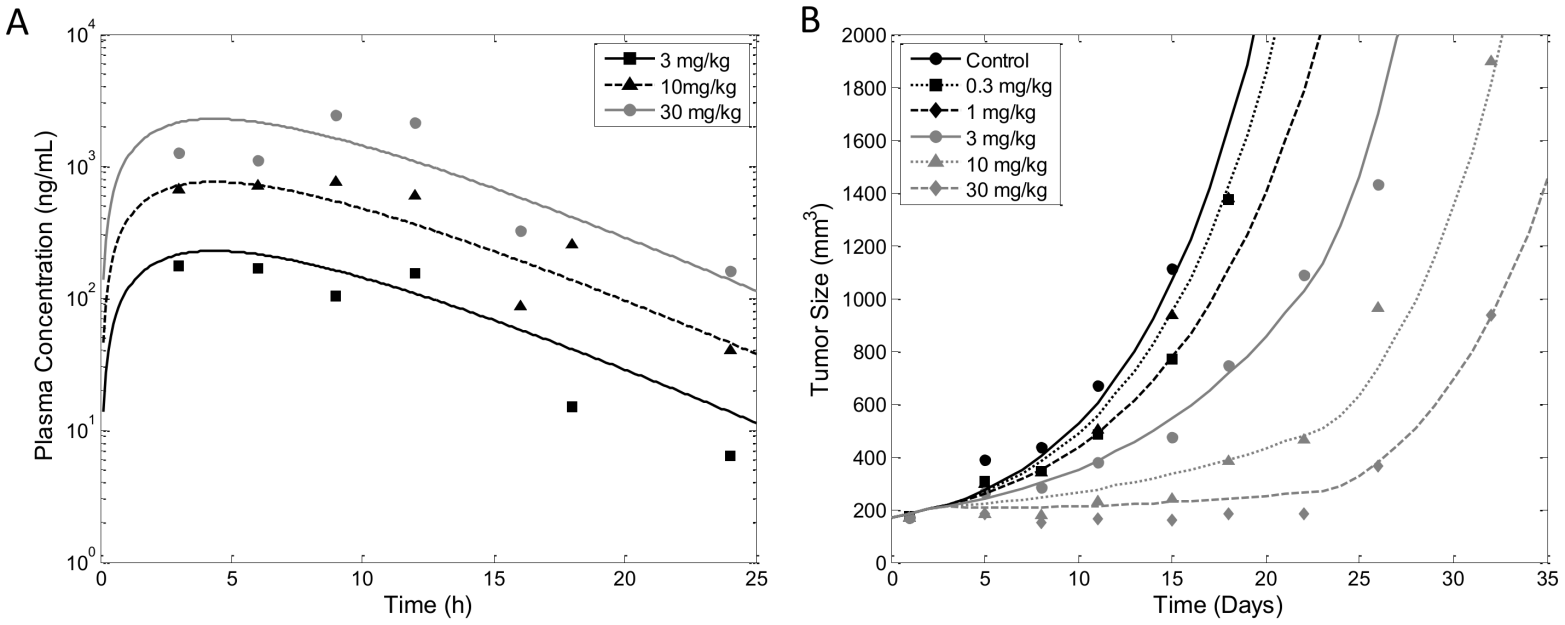
Tumor Growth Inhibition by AZD1208. A) Pharmacokinetic profile of AZD1208 was described using a 1 compartment model with equal values for absorption and elimination rate constants. PK data (symbols) were collected following a single oral administration of AZD1208 at 3, 10 and 30 mg/kg (model prediction in lines). B) Observed (symbol) and lifespan model predicted (lines) tumor growth and inhibition by AZD1208 at 0.3, 1, 3, 10 and 30 mg/kg given orally. Initial tumor volume was fixed to 170 mm^3^, which is the value of the first tumor size measurement.

Data for this case study was provided by AstraZeneca [18]. For the purpose of this case study we will set time according to when the first dose of AZD1208 was administered, which we count as day 1. We began with a compartmental PK analysis after a single oral administration of AZD1208 at 3, 10 and 30 mg/kg and found it well described using a one-compartment PK model with first order absorption as shown in Figure 9A. Parameter values of *k_a_* = 5.52 day^−1^ and *V/F* = 4.86 L/kg were estimated with high precision (Table 5), with the exception of the elimination rate constant, *k_el_*, which was estimated to be same value as *k_a_* and therefore we set *k_el_* to equal *k_a_* value in the final PK model.

**Table 5:** Parameter Estimates for PK Fitting of AZ968.

Parameter	Estimates	Units	CV%
*k_a_*	5.52	1/day	7.48
*V/F*	4.86	L/kg	17.89

After establishing the PK model, we fit the TGI data using the lifespan TGI model. Since AZD1208 is a pan-Pim kinase inhibitor that promotes apoptosis at any stages of cell cycle, the data were analyzed using the non-cycle-specific model. Model fitting of the AZD1208 TGI data is shown in Figure 9B. Interestingly, the tumor data in this study exhibited only exponential growth kinetics. In order to adapt the lifespan TGI model to account for only exponential growth we simply set the tumor threshold, *w_th_*, to a very large value well beyond the observed values of tumor size. Another unique feature of this dataset is the inclusion of saturating doses. To accommodate the dose ranges we replaced the drug potency constant, *k_2_*, in eq. 18 with the *E_max_* model. The process of fitting was the same as for the previous studies, however, in this study the control data were very sparse and the doubling time, *T*, could not be estimated. In order to proceed with the analysis we set *T* = 3 days based on the doubling time reported by the commercial vendor of the MOLM-16 tumor cell used in the study, which was similar to the doubling reported the original study when the cells were initially collected [33]. Doubling times for mouse xenografts have been found to be in the range of 2–8 days, approximately [34]. The remaining parameters were estimated with high precision and are well within biologically feasible ranges (see Table 6). This case study demonstrates the flexibility of the lifespan model to accommodate different types of TGI data while maintaining parameter identifiability and precise estimates of parameter values.

**Table 6:** Parameter Estimates for Lifespan Model fitting of Tumor Growth Inhibition by AZD1208.

Parameter	Estimates	Units	CV%
*T*	3^a^	days	-
*T_A_*	1.92^a^	days	-
*p_0_*	1.57	-	2.17
*k_in0_*	27.8	mm^3^/day	13.5
*E_max_*	0.159	-	5.73
*EC_50_*	182	ng/mL	2.29×10^−2^

a Parameter was fixed.

## Discussion

The lifespan model of TGI presented here is the first mechanistic model to describe tumor growth through the process of cellular division. By using division efficiency as the restriction factor due to tumor burden, the lifespan TGI model is able to account for multi-phasic growth patterns, a critical feature in many models of TGI [8], [9], [35]–[38]. Exploration of the model behavior through simulations, and data fitting capability through case studies have confirmed that our mechanistic approach to modeling TGI is fully capable of describing real experimental data in a biologically relevant context.

Major concerns for development of mechanistic models are the cost of increased number of parameters, and their identifiability. For comparative purposes, we will focus on a well-known TGI model developed by Simeoni and colleagues [11], which is one of the most popular models of TGI. The Simeoni model is a perfect example of modeling parsimony. Its flexibility and robustness allows the model to fully capture tumor growth and tumor growth inhibition data with very few parameters, making it an ideal model for rapid screening of drug libraries. However, the model describes tumor growth empirically through rate kinetics, and utilizes little information about physiological processes in its structural design. The foundation of our lifespan model is the biological mechanism of cancer growth: cell division. Although we introduce more parameters, (four parameters, compared to Simeoni *et al.*'s two parameters), the sensitivity analysis demonstrated that all the parameters are identifiable. Furthermore, the parameters we introduce have much greater biological relevance: *T* is the exact representation of the doubling time of tumor cells; *p_0_* is the division efficiency of the tumor cells at the first observation; and *w_th_* is the tumor size at which the rate of tumor growth changes from exponential to linear. The biological relevance of these parameters can be valuable for inter-species translation, and increase the accuracy of our predictions to human patients. For instance, the doubling time *T* can be directly compared to the doubling time of tumor cells in cancer patients to evaluate any potential in predicting human efficacy of anti-cancer drugs. Appropriate clinical data is required to explore the predictive power of our lifespan TGI model.

The only parameter in the lifespan TGI model that is difficult to interpret is the *k_in0_*, which is the tumor growth rate at the beginning of the experiment. A function describing the tumor growth in the past is necessary due to the use of delay differential equations in our model. By incorporating this parameter, we make the assumption that tumor growth prior to start of the experiment (between t = −*T* to 0) is constant. This is a safe assumption since the doubling time is small in comparison to the duration of the experiment and any tumor growth during that period will be negligible compared to the tumor sizes that will be measured throughout the course of the experiment. It is theoretically feasible to describe the history as a function of time rather than a constant. However, other assumptions will still be necessary, and the implementation of the model becomes very complex and outside the scope of this report.

To incorporate anti-cancer drug mechanism into our lifespan model of TGI, specifically the non-cycle-specific drug effect, we introduced two additional parameters, the drug potency constant *k_2_* and duration of apoptosis, *T_A_*. It should be noted that the drug effect can also be modeled using the Emax function or any other function, but for simplicity and model comparison purposes (reference model uses the same function) we used the simplest drug effect function possible. An additional cell population with a lifespan, *T_A_*, was incorporated to account for the delay in onset of observable drug effect on tumor size, a well reported feature of anti-cancer drug treatment [5]–[7]. This population is generated as a result of anti-cancer drug effect and the lifespan of this population can be interpreted as the time for cell death via apoptosis. Many PKPD models use transit compartments to incorporate delays [9]–[11], [39]. We used the lifespan model to describe the process of cell division, and since transit compartments are approximations of lifespan models, and are equivalent under certain conditions [40], [41], it was natural for us to describe the dying tumor cells using a lifespan model as well.

The mechanistic nature of our lifespan TGI model also allows for incorporation of different anti-cancer drug mechanisms. Although many drugs exhibit the mechanism of action by inducing apoptosis at any stage of the tumor cell life cycle, there are numerous drugs that are cell-cycle-specific [31] and only target cells at specific points in their life cycle. Previous models relied on the time-dependent transition of tumor cells into different drug-sensitivity states [42]. More recent mechanistic models are describing the cell-cycle in more detail [29], [43], [44], however, these models are more complex and require rich data with numerous biomarkers. In our lifespan TGI model we can mechanistically describe a cycle-specific drug action because the model describes changes in tumor size after each round of cell division. After each division cycle, the effect of the drug can be imposed on the tumor cells. And since the time it takes for any cell-cycle stage to repeat itself is the doubling time, this model is applicable for drugs that target different stages of the cell cycle. The application of this cell-cycle-specific drug effect model was demonstrated in Case Study 1, where we examined the effect of paclitaxel on mice xenograft tumors. The model performed well and was able to estimate parameter values with high precision. Interestingly, the model predicted a very low *EC_50_* relative to the plasma concentration. As discussed earlier, this is due to the model equation which only allows the drug to act for a short period of time, during which the drug can affect only a fraction of cells that are sensitive. In other models it is assumed that as soon as the tumor cells are exposed to the drug they are marked for death. The delay for cycle-specific drugs would include not only the time for apoptosis to occur, but also for the time it takes for the cells to become sensitive to the drug. This is not the case in our model, which only exposes the cells to the drug after the doubling time, but this, as demonstrated by the case study, has its drawbacks in the compensatory high estimate of potency. This suggests that cycle-specific drugs must have some means of accumulating in the system. Drug accumulation can be easily modeled using an effect compartment rather than linking the PD to plasma drug concentration. Tumor permeation and clearance can also cause disassociation of predicted drug effect based on plasma drug concentration. Modeling efforts are focused on addressing issues such as tumor vascularization, intra-tumor drug gradients, and tumor heterogeneity, which all can cause discrepancies between intra-tumor and plasma drug PK (see review [45]). However, such modeling efforts require additional data, such as intra-tumor drug PK measurements that we do not have access to. It would be very interesting to combine our lifespan TGI model with a more complex intra-tumor PK model, which would allow for more accurate prediction of efficacy than predictions made based on plasma PK. It should be noted that although this model is able to describe the action of cycle-specific drugs, it is unable to identify the specific cell cycle phase at which the drug becomes active. Inclusion of cell cycle phases are required for cycle identification, and such model is outside the scope of this report.

Although all model parameters are identifiable, the estimation of the doubling time, *T*, and duration of apoptosis, *T_A_*, cannot be accomplished using traditional minimization algorithms. As discussed earlier, this is due to a discontinuity of the model equation with respect to the two parameters. In order to have an estimate of the parameters, a grid search was performed. Although this method is capable of identifying the global minimum, its precision is limited by the run time and number of parameters to be estimated. Furthermore, there are no conventional statistical evaluations available for this approach. The other parameters were estimated using a minimization algorithm after values for *T* and *T_A_* were determined by the grid search method and fixed. Although this is a limitation of the lifespan TGI model, there are options to overcome this issue. One solution is to fix the parameter to *in vitro* values as was done for Case Study 2 with AZD1208 induced TGI. The inclusion of a grid search process greatly adds to the computational time of this model, which can take several hours. This is in addition to the added computation time required for the delay differential equation solver in the algorithmic fitting process, which for the case study presented was more than ten times longer (179 s for lifespan TGI, and 12 s for Simeoni TGI model). Until we can resolve the requirement for a grid search, this model will continue to run much slower by comparison to conventional TGI models.

One of the biggest potential limitations of mechanistic models in general is the need for high resolution data due to their complexity and high number of parameters. The model comparison study showed that although we introduced two additional parameters in our lifespan TGI model compared to the reference TGI model [11], the parameters in our model were estimated with high precision, and that the model was able to describe the data well compared to the reference model. Furthermore, the estimated values of equivalent parameters between the two models were in very good concordance. This comparative study demonstrates that our model not only offers a mechanistic approach to describing tumor growth, it also has comparable robustness to the reference model when applied to real experimental data. Although the model comparison was done with real data, the cost of the extra parameters could become apparent with sparse data [46]. Even though our model is based on biological processes, some of the concepts we introduce are difficult if not impossible to confirm with current technologies. For instance the proliferation efficiency, described by the *p(w)* function, cannot be confirmed as it is not feasible to track *in vivo* cell division in real time. Certain model parameters, such as the initial tumor growth rate, *kin_0_*, the doubling time, *T*, and time for tumor death, *T_A_*, are all values that cannot be confirmed *in vivo*. Therefore the accuracy of the model and its parameter estimates must be taken with caution. One of the greatest limitations of many TGI models is the oversimplification of the cellular composition of tumors. Tumor heterogeneity has been widely observed both clinically and experimentally [47]–[50]. However, in order to characterize heterogeneous cell populations, additional biomarkers are required. Although it is very feasible to quantify mixed cell populations through flow cytometry or histological techniques, such experiments are invasive and would disrupt accurate collection of continuous tumor growth data. Mixed cell population modeling would also require additional model parameters. In regards to model parsimony and given the nature of the available data, many established TGI models have relied on the simplification of the tumor cellular composition [8], [10], [11].

The lifespan TGI model presented in this report is a novel mechanistic approach to modeling tumor growth and tumor growth inhibition by anti-cancer drugs. We have demonstrated that the model has the versatility to address different tumor growth kinetics and different drug mechanisms of action, and the robustness to provide precise parameter estimates from experimental data. Most importantly, the mechanistic nature of the model and the parameters has biological relevance, which can have real implications in inter-species predictions.

## Supporting Information

Appendix S1 **Derivation of model equations.**
(DOCX)

Appendix S2 **Explicit solution to the tumor growth model with constant cell division efficiency.**
(DOCX)

Appendix S3 **Calculation of the slope of the tumor size vs. time curve.**
(DOCX)

Material S1 **Non-cycle-specific model run script.**
(M) Click here for additional data file.

Material S2 **Non-cycle-specific model file.**
(M) Click here for additional data file.

Material S3 **Non-cycle-specific model history file.**
(M) Click here for additional data file.

Material S4 **Cycle-specific model run script.**
(M) Click here for additional data file.

Material S5 **Cycle-specific model file.**
(M) Click here for additional data file.

Material S6 **Cycle-specific model history file.**
(M) Click here for additional data file.
