# Supplementary material for: Lifespan Based Pharmacokinetic-Pharmacodynamic Model of Tumor Growth Inhibition by Anticancer Therapeutics
Source: PLoS One. 2014 Oct 21;9(10):e109747. doi: 10.1371/journal.pone.0109747 (PMC4204849; doi:10.1371/journal.pone.0109747)
Supplement: Appendix S1 — Derivation of model equations. (DOCX) [file pone.0109747.s001.docx]

**Appendix S1**

***Derivation of model equations***

For sake of space we will only consider the model with the chemotherapeutic effect. To account for the model without a drug one can simply set *E(C(t))* = 0 and *A(t)* = 0 for all possible *t* values in the following calculations. First we derive the eq. 30 describing *k_in_(t)*. For 0 < *t* < *T*, *C(t-T)* = 0 and *k_in_(t-T)* = *k_in0_*. Therefore eq. 29 implies that:

| $k_{in}(t)=k_{in0}\cdot p(w(t))$ | A 1 |
| --- | --- |

Applying eq. 30 for the interval 0 < *t* < *T* yields:

| $k_{in}(t)=\frac{k_{in0}}{1-E(C(t))}\cdot(1-E(C(t)))\cdot p(w(t))$ | A 2 |
| --- | --- |

So, A 1 and A 2 are equal. To show that eq. 30 holds for all *t* > 0, we use mathematical induction to derive eq. 30 for 0 < *t* < *(j+1)·T*, assuming eq. 30 holds true for 0 < *t* < *jT*. Again, for *jT* ≤ *t* < *(j+1)T* eq. 29 yields:

| $\begin{matrix} k_{in}(t)=p(w(t))\cdot(1-E(C(t-T)))\cdot\frac{k_{in0}}{1-E(C(t-T))}\cdot\\ \prod_{i=0}^{j-1} (1-E(C(t-T-iT)))\cdot p(w(t-T-iT)) \\ =k_{in0}\cdot p(w(t))\cdot\prod_{i=1}^{j} (1-E(C(t-iT)))\cdot p(w(t-iT)) \end{matrix}$ | A 3 |
| --- | --- |

On the other hand, applying eq. 30 for *jT* ≤ *t* < *(j+1)T* results in:

|  | A 4 |
| --- | --- |

Since equations A 3 and A 4 are equal, the proof of eq. 30 is complete.

Next, we will apply eq. 30, to calculate *k_out_(t)* using eq. 28 and obtain the model equation as outlined by eq. 1. Consider times 0 < *t* < *T*. Then:

| $k_{in}(t)-k_{out}(t)=k_{in}(t)\cdot(1-E(C(t))-k_{in0}=k_{in0}\cdot((1-E(C(t))\cdot p(w(t))-1)$ | A 5 |
| --- | --- |

which coincides with eq. 31, since *INT(t/T)* = 0.

For times *t* ≥ *T* :

| $\begin{matrix} k_{in}(t)-k_{out}(t)=k_{in}(t)\cdot(1-E(C(t))-(1-E(C(t-T)))\cdot k_{in}(t-T)= \\ k_{in0}\cdot\prod_{i=0}^{INT(t/T)} (1-E(C(t-iT)))\cdot p(w(t-iT)) \\ -k_{in0}\cdot\prod_{i=0}^{INT(t/T)-1} (1-E(C(t-T-iT)))\cdot p(w(t-T-iT))= \\ k_{in0}\cdot\prod_{i=0}^{INT(t/T)} (1-E(C(t-iT)))\cdot p(w(t-iT))-k_{in0}\cdot\prod_{i=1}^{INT(t/T)} (1-E(C(t-iT)))\cdot p(w(t-iT)) \\ =k_{in0}\cdot\left(1-\frac{1}{(1-E(C(t)))\cdot p(w(t))}\right)\cdot\prod_{i=0}^{INT(t/T)} (1-E(C(t-iT)))\cdot p(w(t-iT)) \end{matrix}$ | A 6 |
| --- | --- |

which collapses to eq. 31 when the term in front of the product is further transformed. This completes proof of eq. 31.
