# Supplementary material for: Lifespan Based Pharmacokinetic-Pharmacodynamic Model of Tumor Growth Inhibition by Anticancer Therapeutics
Source: PLoS One. 2014 Oct 21;9(10):e109747. doi: 10.1371/journal.pone.0109747 (PMC4204849; doi:10.1371/journal.pone.0109747)
Supplement: Appendix S2 — Explicit solution to the tumor growth model with constant cell division efficiency. (DOCX) [file pone.0109747.s002.docx]

**Appendix S2**

***Explicit solution to the tumor growth model with constant cell division efficiency***

Integration of both sides of eq. 10 from 0 to *t* yields:

|  | B 1 |
| --- | --- |

where the change of variables *s* = *z/T* was done in the integral in B 1. The integral can be divided into two integrals:

| $\int_{0}^{t/T} p_{0}^{INT(s)}ds=\int_{0}^{INT(t/T)} p_{0}^{INT(s)}ds+\int_{INT(t/T)}^{t/T} p_{0}^{INT(s)}ds$ | B 2 |
| --- | --- |

where the second integral becomes:

| $\int_{INT(t/T)}^{t/T} p_{0}^{INT(s)}ds=p_{0}^{INT(t/T)}\cdot(t/T-INT(t/T))$ , for 0 < *t* < *T* | B 3 |
| --- | --- |

Given the condition of *INT(t/T)* = 0, equation B 3 becomes:

| $\int_{0}^{t/T} p_{0}^{INT(s)}ds=t/T$ , for 0< t < T | B 4 |
| --- | --- |

and consequently:

| $w(t)=w_{0}+k_{in0}\cdot T\cdot(p_{0}-1)\cdot t/T$ , for 0 < t < T | B 5 |
| --- | --- |

which equals to eq. 11.

For *t* ≥ *T* the first integral becomes:

| $\int_{0}^{INT(t/T)} p_{0}^{INT(s)}ds=\sum_{i=0}^{INT(t/T)-1} \int_{i}^{i+1} p_{0}^{INT(s)}ds=\sum_{i=0}^{INT(t/T)-1} p_{0}^{i}=\frac{p_{0}^{INT(t/T)}-1}{p_{0}-1}$ | B 6 |
| --- | --- |

Combining B 3and B 6 yields:

| $\begin{matrix} w(t)=w_{0}+k_{in0}\cdot T\cdot(p_{0}-1)\cdot\frac{p_{0}^{INT(t/T)}-1}{p_{0}-1}+k_{in0}\cdot T\cdot(p_{0}-1)\cdot p_{0}^{INT(t/T)}\cdot(t/T-INT(t/T)) \\ =w_{0}+k_{in0}\cdot T\cdot(p_{0}^{INT(t/T)}-1+(p_{0}-1)\cdot p_{0}^{INT(t/T)}\cdot(t/T-INT(t/T))) \end{matrix}$ | B 7 |
| --- | --- |

which equals eq. 11. This completes the proof of eq. 11
