# Supplementary material for: Lifespan Based Pharmacokinetic-Pharmacodynamic Model of Tumor Growth Inhibition by Anticancer Therapeutics
Source: PLoS One. 2014 Oct 21;9(10):e109747. doi: 10.1371/journal.pone.0109747 (PMC4204849; doi:10.1371/journal.pone.0109747)
Supplement: Appendix S3 — Calculation of the slope of the tumor size vs. time curve. (DOCX) [file pone.0109747.s003.docx]

**Appendix S3**

***Calculation of the slope of the tumor size vs. time curve***

The curve *w(t)* vs. *t* exhibits a linear growth for large time. Calculating its slope in general is difficult. We will provide an approximation under an assumption that *T* is small compared to all other time scales in the model (*T*→0). The calculation will require three steps. First, we will show that *w(t)* is a strictly increasing function of time. Second, *w(t)* increases to infinity as *t* goes to infinity. Last, we will calculate the slope of *w(t)* for large times.

Notice that the equation describing tumor growth eq. 8 can be written for *t* > *T* in the following form:

| $\frac{dw}{dt}=k_{in0}\cdot(p(w(t))-1)\cdot\prod_{i=1}^{INT(t/T)} p(w(t-iT))$ | C 1 |
| --- | --- |

To show that:

| $\frac{dw}{dt}>0$ , for all *t* > 0 | C 2 |
| --- | --- |

it suffices to observe that *p(w)* > 1 all *w* > 0. Hence the right hand side of equation C 1 is positive. Consequently, *w(t)* is a strictly increasing function of time. Also:

| $\frac{dw}{dt}\geq k_{in0}\cdot(p(w(t))-1)$ , for t >0 | C 3 |
| --- | --- |

Hence:

| $\frac{1}{(p(w(t))-1)}\cdot\frac{dw}{dt}\geq k_{in0}$ | C 4 |
| --- | --- |

Integration of both sides of C 4 over time from 0 to *t* yields:

| $\frac{1}{p(w(t))-1}-\frac{1}{p(w_{0})-1)}\geq k_{in0}\cdot t$ | C 5 |
| --- | --- |

C 5 implies that 1/(*p(w(t))*-1) approaches infinity as *t* → ∞. This means that *p(w(t))* → 1, and subsequently *w(t)* → ∞, as *t* → ∞.

Further calculation will be carried utilizing the following relationship:

| $\prod_{i=1}^{INT(t/T)} p(w(t-iT))\sim\exp\left(\frac{1}{T}\cdot\int_{0}^{t} \ln\left(p(w(\tau))\right)d\tau\right)$, as *T* → 0 | C 6 |
| --- | --- |

The symbol “~” describes the asymptotic behavior of the left hand side of C 6 for small *T* and is defined as follows (based on concepts outlined in [[1](#_ENREF_1)]):

| , as *x* →*x_0_* ⇔ ,as *x* →*x_0_* | C 7 |
| --- | --- |

Notice that:

| $\prod_{i=1}^{INT(t/T)} p(w(t-iT))=\exp\left(\frac{1}{T}\cdot\sum_{i=1}^{INT(t/T)} \ln\left(p(w(t-iT))\right)\cdot T\right)$ | C 8 |
| --- | --- |

The definition of the Riemann integral [[2](#_ENREF_2)] implies:

| $\sum_{i=1}^{INT(t/T)} \ln\left(p(w(t-iT))\right)\cdot T\to\int_{0}^{t} \ln\left(p(w(t-s))\right)ds$ , as *T* → 0 | C 9 |
| --- | --- |

C 9 and definition C 7 guarantee that:

| $\prod_{i=1}^{INT(t/T)} p(w(t-iT))\sim\exp\left(\frac{1}{T}\cdot\int_{0}^{t} \ln\left(p(w(t-s))\right)ds\right)$ , as *T* → 0 | C 10 |
| --- | --- |

Changing variables *τ* = *t* - *s* in the integral in C 10 results in C 6. From here on we will suppress the notation *T* → 0. Consequently, C 1 implies that:

| $\frac{dw}{dt}\sim k_{in0}\cdot\left(p(w(t))-1\right)\cdot\exp\left(\frac{1}{T}\cdot\int_{0}^{t} \ln\left(p(w(\tau))\right)d\tau\right)$ | C 11 |
| --- | --- |

In order for $\frac{dw}{dt}$to converge to a positive value, the integral in C 11 must approach infinity for large times:

| $\int_{0}^{t} \ln\left(p(w(\tau))\right)d\tau\to\infty$ , as *t* → ∞ | C 12 |
| --- | --- |

To prove C 12 by leading to a contradiction, let us assume that:

| $\int_{0}^{t} \ln\left(p(w(\tau))\right)d\tau\to a>0$ , as *t* → ∞ | C 13 |
| --- | --- |

Consequently:

| $\frac{1}{p(w(t))-1}\cdot\frac{dw}{dt}\sim k_{in0}\cdot\exp\left(\frac{a}{T}\right)$ , as *t* → ∞ | C 14 |
| --- | --- |

Since:

| $\frac{1}{p(w(t))-1}\sim\frac{w(t)}{(p_{0}-1)w_{th}}$ , as *t* → ∞ | C 15 |
| --- | --- |

Then:

| $\int_{0}^{t} \frac{1}{p(w(s))-1}\cdot\frac{dw}{ds}ds\sim\int_{0}^{t} \frac{w(t)}{(p_{0}-1)w_{th}}\cdot\frac{dw}{ds}ds=\frac{w(t)^{2}-w_{0}^{2}}{2\cdot(p_{0}-1)\cdot w_{th}}$ | C 16 |
| --- | --- |

Equation C 14 implies:

| $\frac{w(t)^{2}-w_{0}^{2}}{2\cdot(p_{0}-1)\cdot w_{th}}\sim k_{in0}\exp\left(\frac{a}{T}\right)\cdot t$ | C 17 |
| --- | --- |

And:

| $w(t)\sim\sqrt{w_{0}^{2}+2\cdot(p_{0}-1)\cdot w_{th}\cdot k_{in0}\exp\left(\frac{a}{T}\right)\cdot t}$ | C 18 |
| --- | --- |

Subsequently:

$\ln\left(p\left(w(t)\right)\right)\sim\ln\left(p\left(\sqrt{w_{0}^{2}+2\cdot(p_{0}-1)\cdot w_{th}\cdot k_{in0}\cdot\exp\left(\frac{a}{T}\right)\cdot t}\right)\right)\sim\frac{2\cdot(p_{0}-1)\cdot w_{th}}{\sqrt{w_{0}^{2}+2\cdot(p_{0}-1)\cdot w_{th}\cdot k_{in0}\cdot\exp\left(\frac{a}{T}\right)\cdot t}}$C 19

Hence:

| $\int_{0}^{t} \ln\left(p\left(w(s)\right)\right)ds\sim\int_{0}^{t} \frac{2\cdot(p_{0}-1)\cdot w_{th}ds}{\sqrt{w_{0}^{2}+2\cdot(p_{0}-1)\cdot w_{th}\cdot k_{in0}\cdot\exp\left(\frac{a}{T}\right)s}}$ | C 20 |
| --- | --- |

Since:

| $\int_{0}^{t} \frac{2\cdot(p_{0}-1)\cdot w_{th}ds}{\sqrt{w_{0}^{2}+2\cdot(p_{0}-1)\cdot w_{th}\cdot k_{in0}\cdot\exp\left(\frac{a}{T}\right)s}}\to\infty$ , as *t* → ∞ | C 21 |
| --- | --- |

Then:

| $\int_{0}^{t} \ln\left(p\left(w(s)\right)\right)ds\to\infty$ , as *t* → ∞ | C 22 |
| --- | --- |

Now that C 22 contradicts C 13 the proof of equation C 12 is complete.

To calculate the limit $\frac{dw}{dt}$as *t* →∞, we will transform the right hand side of C 11 to the following form:

| $\lim_{t\to\infty} \frac{k_{in0}\cdot\exp\left(\frac{1}{T}\cdot\int_{0}^{t} \ln\left(p(w(\tau))\right)d\tau\right)}{\frac{1}{\left(p(w(t))-1\right)}}=k_{in0}\cdot(p_{0}-1)\cdot\lim_{t\to\infty} \frac{\exp\left(\frac{1}{T}\cdot\int_{0}^{t} \ln\left(p(w(\tau))\right)d\tau\right)}{\frac{w(t)}{w_{th}}}$ | C 23 |
| --- | --- |

The l’Hospital rule [[2](#_ENREF_2)] implies:

| $\lim_{t\to\infty} \frac{\exp\left(\frac{1}{T}\cdot\int_{0}^{t} \ln\left(p(w(\tau))\right)d\tau\right)}{\frac{w(t)}{w_{th}}}=\lim_{t\to\infty} \frac{\frac{1}{T}\cdot\ln\left(p(w(t))\right)\cdot\exp\left(\frac{1}{T}\cdot\int_{0}^{t} \ln\left(p(w(\tau))\right)d\tau\right)}{\frac{1}{w_{th}}\cdot\frac{dw(t)}{dt}}$ | C 24 |
| --- | --- |

From C 11:

| $\frac{\exp\left(\frac{1}{T}\cdot\int_{0}^{t} \ln\left(p(w(\tau))\right)d\tau\right)}{\frac{dw}{dt}}\sim\frac{1}{k_{in0}\cdot p(w(t))-1}$ | C 25 |
| --- | --- |

Combining equations C 23 to C 25

| $\lim_{t\to\infty} \frac{dw}{dt}=\frac{(p_{0}-1)\cdot w_{th}}{T}\cdot\lim_{t\to\infty} \frac{\ln\left(p(w(t))\right)}{p(w(t))-1}=\frac{(p_{0}-1)\cdot w_{th}}{T}$ | C 26 |
| --- | --- |

This completes the calculation of the slope of *w(t)* vs. *t* curve for large times.

**References**

1. Erdélyi A (1956) Asymptotic Expansions. New York: Dover Publications. p.11 p.

2. Hunt RA (1994) Calculus: HarperCollins College Publishers.
